# Transcript Levels of Major Interleukins in Relation to the Clinicopathological Profile of Patients with Tuberculous Intervertebral Discs and Healthy Controls

**DOI:** 10.1371/journal.pone.0101324

**Published:** 2014-06-27

**Authors:** Chong Liu, Xinli Zhan, Zengming Xiao, Qie Fan, Li Deng, Mingxing Cui, Chunxiang Xiong, Jingbo Xue, Xiangtao Xie

**Affiliations:** Spine and Osteopathy Ward, The First Affiliated Hospital of Guangxi Medical University, Nanning, Guangxi, People’s Republic of China; University of Cape Town, South Africa

## Abstract

**Objectives:**

The purpose of the present study was to simultaneously examine the transcript levels of a large number of interleukins (ILs; IL-9, IL-10, IL-12, IL-13, IL-16, IL-17, IL-18, IL-26, and IL-27) and investigate their correlation with the clinicopathological profiles of patients with tuberculous intervertebral discs.

**Methods:**

Clinical data were collected from 150 patients participating in the study from January 2013 to December 2013. mRNA expression levels in 70 tuberculous, 70 herniated, and 10 control intervertebral disc specimens were determined by real-time polymerase chain reaction.

**Results:**

IL-10, IL-16, IL-17, IL-18, and IL-27 displayed stronger expression in tuberculous spinal disc tissue than in normal intervertebral disc tissue (P<0.05). Our results illustrated multiple correlations among IL-10, IL-16, IL-17, IL-18, and IL-27 mRNA expression in tuberculous samples. Smoking habits were found to have a positive correlation with IL-17 transcript levels and a negative correlation with IL-10 transcript levels (P<0.05). Pain intensity, symptom duration, C-reactive protein levels, and the erythrocyte sedimentation rate exhibited multiple correlations with the transcript levels of several ILs (P<0.05).

**Conclusions:**

The experimental data imply a double-sided effect on the activity of ILs in tuberculous spinal intervertebral discs, suggesting that they may be involved in intervertebral discs destruction. Our findings also suggest that smoking may affect the intervertebral discs destruction process of spinal tuberculosis. However, further studies are necessary to elucidate the exact role of ILs in the intervertebral discs destruction process of spinal tuberculosis.

## Introduction

Tuberculosis (TB) represents a challenging public health problem across the world. According to the World Health Organization, one-third of the world’s population is believed to be infected with the causative bacterium *Mycobacterium tuberculosis*. Spinal TB is the most common form of bone TB in developing countries [1]. Numerous articles have been published on spinal TB in recent decades [2]–[4]. Spinal TB usually affects the intervertebral discs, leading to the destruction of spinal stabilization, adjacent vertebral bodies, and surrounding soft tissue. Although spinal TB is common, little information is available on the inflammatory and immune mechanisms involved in its development. In particular, it is unclear which cells and mediators are involved in the intervertebral disc destruction processes and whether resident immunocompetent cells orchestrate the development of an inflammatory response. The interleukin (IL) family plays important roles in inflammatory and immune responses to TB [5]–[7]. The role of such factors in the pathogenesis of intervertebral disc tuberculosis destruction is particularly deserving of elucidation.

The ILs comprise a large group of immunomodulatory proteins that elicit a wide variety of responses in cells and tissues. ILs can exert both inflammatory and anti-inflammatory effects. A few members act as chemoattractants for helper T cells, paralleling the actions of chemokines. Others are intimately involved in the cellular response to viral pathogens, making them akin to interferons (IFNs). ILs are extremely important mediators of the physiological response to infection, and they also contribute significantly to the pathophysiology of a wide range of disorders [8]. As such, they represent a group of proteins with potential importance as therapeutic targets.

IFN-gamma, TNF, and matrix metalloproteinases are important inflammatory markers in the pathogenesis of TB. Some published reports investigated their role in the pathogenesis of TB [9]–[12]. However, there are few published data on the role of ILs in the pathogenesis of tuberculous intervertebral discs, particularly in human samples. Based on the aforementioned background, the interleukins (ILs; IL-9, IL-10, IL-12, IL-13, IL-16, IL-17, IL-18, IL-26, and IL-27) selected in this study were chosen because they are the most commonly studied members of the IL family [13]–[20]. ILs are assigned to each family based on sequence homology and receptor chain similarities or functional properties. Members of the major subgroups of ILs were included in the analysis, e.g., the IL-1 family (IL-18), the common γ chain family (IL-9), the IL-10 family (IL-10, IL-26), the IL-12 family (IL-12, IL-27), the Th2-like cytokines (IL-13), the ILs with chemokine activity (IL-16), and the IL-17 family (IL-17). Therefore, a quantitative molecular analysis of the transcript levels of a large number of ILs (IL-9, IL-10, IL-12, IL-13, IL-16, IL-17, IL-18, IL-26, and IL-27) will provide insight into the molecular mechanism that regulates the expression of ILs in human tuberculous intervertebral discs.

Nicotine is a major component of cigarette smoke. However, the effects of nicotine on inflammatory and immune cells are incompletely characterized with conflicting conclusions. One set of studies has provided evidence that nicotine promotes inflammation [21], On the contrary, another set of studies has shown that nicotine is a key mediator of the anti-inflammatory pathway [22], and that nicotine suppresses the activity of immune cells [23]. Thus, both pro-inflammatory and anti-inflammatory activities have been attributed to nicotine. However, the precise immunological changes in tuberculous spinal intervertebral discs induced by nicotine have not been fully defined. There are few published data on the role of nicotine in the pathogenesis of tuberculous intervertebral discs. Therefore, a quantitative molecular analysis of the ILs correlated with smoking habits will provide insight into the molecular mechanism that nicotine in the processes of intervertebral disc destruction in spinal tuberculosis.

Therefore, the aim of our study was to elucidate the mRNA expression profile of ILs (IL-9, IL-10, IL-12, IL-13, IL-16, IL-17, IL-18, IL-26, and IL-27) in tuberculous, herniated, and control intervertebral disc specimens. In addition, this study analyzed the correlation of IL transcript levels with the clinicopathological profiles of patients with tuberculous intervertebral discs.

## Materials and Methods

### Tissue samples

All samples were obtained with written informed consent from patients or relatives. This study was approved by the appropriate ethics committee of Guangxi province (China) and therefore was performed in accordance with the ethical standards outlined in the 1964 Declaration of Helsinki and its later amendments. Tuberculous disc tissue samples were obtained from 70 patients who underwent surgery for spinal TB in our hospital from January 1, 2013 to December 30, 2013. These patients were assigned to the TB group. All patients displayed symptoms of spinal TB, such as moderate fever, weakness, back pain, and paraparesis. The diagnosis was established by performing a hematological examination, the Mantoux tuberculin skin test, biopsy and histopathological investigation, and imaging examinations including radiography, computed tomography), and magnetic resonance imaging. Patients with acquired immune deficiency syndrome, tumors, and ankylosing spondylitis were excluded from the study. After excision, two experienced pathologists examined the tissue samples. Samples were obtained after surgery, immediately frozen in liquid nitrogen, and maintained at −80°C until RNA extraction. Seventy herniated disc specimens were also collected from 70 patients who underwent surgery for intervertebral disc herniation. These patients were included in the ID herniation group. Ten fresh human cadaver intervertebral disc specimens were assigned to the control group. The specimens were obtained within 10 h after death. Patients with spinal diseases (e.g., intervertebral disc herniation, spinal TB) were excluded from the control group. The following data were collected for the three groups: age, gender, employment, smoking habits, pain intensity (visual analog scale [VAS] score), level of intervertebral disc herniation, duration of symptoms, C-reactive protein (CRP) levels, and erythrocyte sedimentation rates (ESRs). All relevant data for the three groups are listed in Table 1. There were no significant differences for the data among the three groups (P>0.29).

**Table 1:** Patient clinical characteristics.

Characteristic	TB group	ID herniation group	Control group
**Sex**			
** Male/female**	31/39	34/36	5/5
**Age(year)**			
** Mean(range)**	39(19–62)	42(22–63)	41(22–68)
**Employment**			
** Heavy/light**	34/36	39/31	6/4
**Smoking habits**			
** Smokers/non-smokers**	36/34	33/37	5/5
** <10 cigarettes per day**	14	13	**-**
** 10–20 cigarettes per day**	15	13	**-**
** >20 cigarettes per day**	7	7	**-**
**Level of ID herniation**			
** C2-T1**	8	12	3
** T1-L1**	34	9	3
** L1-S1**	28	49	4
**Pain intensity(VAS)**			
** 0–4**	15	19	**-**
** 5–7**	25	25	**-**
** 8–10**	30	26	**-**
**Duration of symptoms**			
** <3 Months**	13	14	**-**
** 3–12 Months**	25	32	**-**
** >12 Months**	32	24	**-**
**CRP(mg/L)**			
** <10**	11	17	**-**
** 10–30**	22	28	**-**
** >30**	37	25	**-**
**ESR(mm/h)**			
** <20**	13	40	-
** 20–40**	15	30	-
** >40**	42	0	-

### RNA extraction and preparation of cDNA

Total RNA was isolated using TRIzol (Life Technologies, USA) according to the manufacturers’ instructions. The total RNA concentration and quality were measured by a Nanodrop2000 micro-volume spectrophotometer (Thermo Scientific, USA) using absorbance measurements. RNA integrity was analyzed by 2% agarose gel electrophoresis and staining with ethidium bromide.

### Real-time PCR

First-strand cDNA was synthesized from 3000 ng of total RNA using the PrimeScript RT reagent Kit with gDNA Eraser (TaKaRa, Japan) as instructed by the manufacturers. Real-time PCR (RT-PCR) was performed on an ABI7500 real-time PCR system (Life Technologies, USA). The RT-PCR mixture consisted of 10 µl of FastStart Universal SYBR Green Master (Roche, Germany), 1.2 µl of each upstream primer, 7.8 µl of PCR-grade water, and 1 µl of cDNA. The PCR thermal cycling conditions were as follows: 10 min at 95°C followed by 15 s at 95°C (40 cycles) and 1 min at 60°C. To create the RT-PCR standard, beta-actin was used as the internal control. The primer sequences used are shown in Table 2, and they were designed using the UPL Assay Design Centre web service. All RT-PCR products were electrophoresed on 2% agarose gels and visualized using ethidium bromide under a UV light transilluminator. IL transcript levels were calculated using the 2^−△△Ct^ method [24]. All RT-PCR procedures were repeated in triplicates. The results were analyzed using ABI 7500 real-time PCR software version 2.0.1.

**Table 2:** Primer sequences used for quantitative real-time RT-PCR.

Gene	Primer sequence(5′–3′)	Annealing temperature (°C)	Product Size(bp)
IL-9	CTCTGTTTGGGCATTCCCTCT	58	95
	GGGTATCTTGTTTGCATGGTGG		
IL-10	AAAATCGGATCTGGGGCTCT	56	152
	TGGGCTTCTTTCTAAATCGTTC		
IL-12	TCAGAGGGGACAACAAGGAGT	56	119
	CTTGAGCTTGTGAACGGCAT		
IL-13	TTCCAGCTTGCATGTCCGA	58	212
	TAACCCTCCTTCCCGCCTAC		
IL-16	GCTTCTACGGTGTATGGTTCTGT	55	101
	CTCAATCTGCCGCATCACTC		
IL-17	AGATTACTACAACCGATCCACCT	56	151
	GGGGACAGAGTTCATGTGGTA		
IL-18	TCGGGAAGAGGAAAGGAACC	57	80
	GCCATCTTTATTCCTGCGACA		
IL-26	GCTGTTAGTCACTCTGTCTCTTG	55	85
	GGACAATGTTCCCCTTGGGTA		
IL-27	TTTGCGGAATCTCACCTGC	57	83
	TGGAAGGTCAGGGAAACATCA		
Beta-actin	CGAGCACAGAGCCTCGCCTT	60	284
	ATGCCGTGCTCGATGGGGTA		

### Statistical analysis

The Kolmogorov-Smirnov one-sample test was employed to assess the normality of the distribution of the mRNA expression values for the genes studied. Accordingly, the nonparametric test was used to compare the mRNA expression of the ILs in the three groups as well as in groups with different clinicopathological features. The pairwise IL mRNA correlations in the TB and ID herniation groups were evaluated with the Spearman rank correlation test. Statistical analysis was performed using SPSS 16.0 (SPSS, Inc., Chicago, IL, USA). P<0.05 (two-tailed) was considered statistically significant.

## Results

### IL mRNA expression

**Figure 1:**
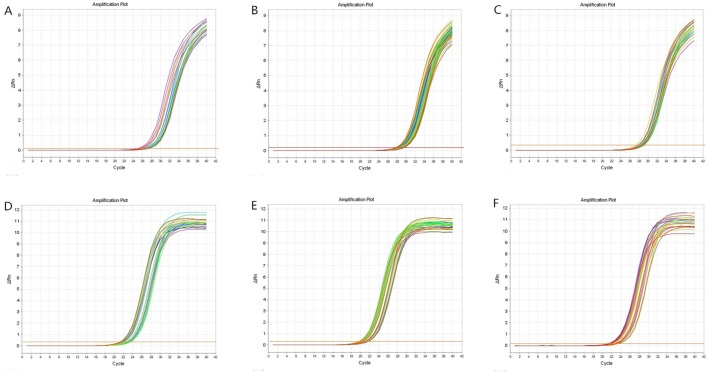
Amplification curves. A: IL-10, the Ct value was within the range of 24–27; B: IL-12, the Ct value was within the range of 26–28; C: IL-16, the Ct value was within the range of 26–28; D: IL-18, the Ct value was within the range of 20–22; E: IL-17, the Ct value was within the range of 20–22; and F: IL-27, the Ct value was within the range of 21–23.

**Figure 2:**
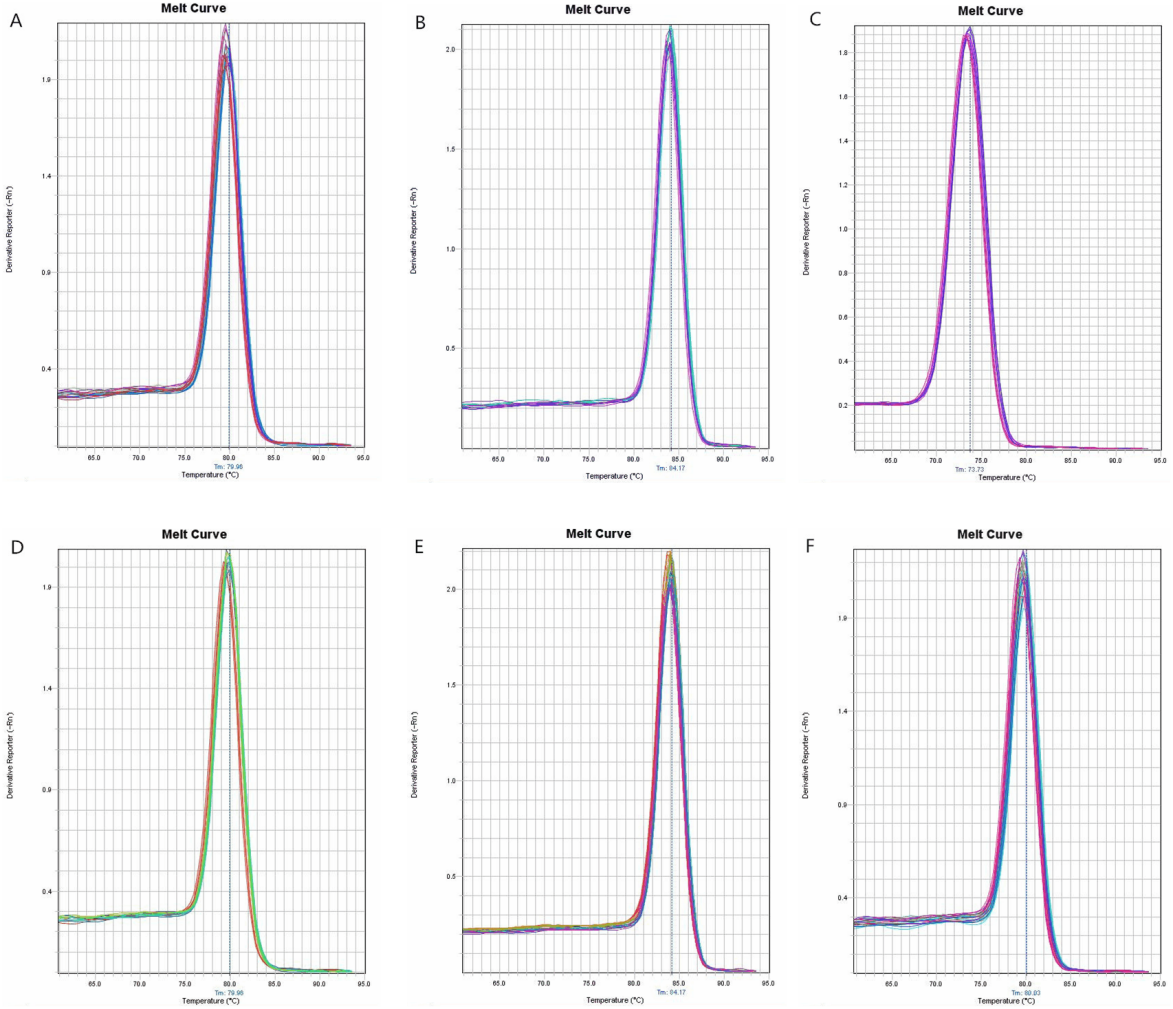
Melting curves. A: IL-10, the specific melting temperature was 79.96°C; B: IL-12, the specific melting temperature was 84.17°C; C: IL-16, the specific melting temper-ature was 73.73°C; D: IL-17, the specific melting temperature was 79.96°C; E: IL-18, the specific melting temperature was 84.87°C; and F: IL-27, the specific melting temperature was 80.03°C.

**Figure 3:**
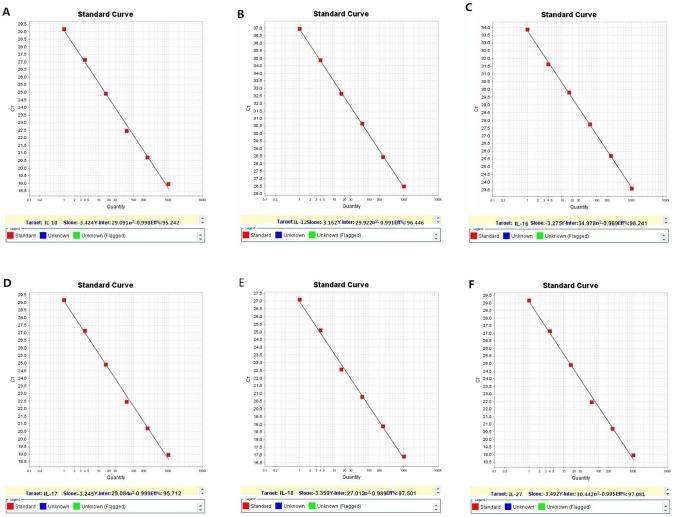
Standard curves. A: IL-10, the efficiency was 95.242% and the correlation coefficient was 0.998; B: IL-12, the efficiency was 96.446% and the correlation coefficient was 0.991; C: IL-16, the efficiency was 98.241% and the correlation coefficient was 0.989; D: IL-17, the efficiency was 95.712% and the correlation coefficient was 0.999; E: IL-18, the efficiency was 97.501% and the correlation coefficient was 0.989; and F: IL-27, the efficiency was 97.081% and the correlation coefficient was 0.995.

IL-9, IL-13, and IL-26 were not amplified in the three groups. The IL-10, IL12, IL-16, IL-17, IL-18, and IL-27 amplification curves revealed that the cycle threshold (Ct) values were within an acceptable range (Figure 1). The melting curves illustrated the specificity of the primers (Figure 2). The standard curves were plotted as the Ct versus the concentration of the total RNA from serial dilutions (Figure 3).

IL mRNA was not detected in all tissue samples, but the detection rates of the ILs examined in this study did not differ significantly among the three groups (Table 3). Significantly higher mRNA expression in the TB group than in the control group was detected for IL-10, IL-16, IL-17, IL-18, and IL-27 (P<0.05). The mRNA expression of IL-10, IL-16, IL-18, and IL-27 mRNA was higher in the TB group than in the ID herniation group (P<0.05) (Table 4). Our data also demonstrated that patients with more severe disease had significantly higher IL-16, IL-17, and IL-18 expression and significantly lower IL-10 and IL-27 expression (Table 5).

**Table 3:** Detected mRNA expression of ILs in the three groups.

Gene	TB group %(N)	ID herniation %(N)	Control group %(N)	P value
**IL-10**	87(61/70)	87(61/70)	80(8/10)	ns
**IL-12**	89(62/70)	91(64/70)	90(9/10)	ns
**IL-16**	90(63/70)	86(60/70)	90(9/10)	ns
**IL-17**	84(59/70)	89(62/70)	90(9/10)	ns
**IL-18**	89(65/70)	90(63/70)	80(8/10)	ns
**IL-27**	87(61/70)	87(61/70)	70(7/10)	ns

ns = not significant.

**Table 4:** The mRNA expression of the ILs in the three groups.

Gene	TB group (n = 70)	ID herniation (n = 70)	Control group (n = 10)
**IL-10**	6.52±1.12^a,b^	2.32±0.38^c^	2.15±0.43^c^
**IL-12**	0.78±0.12	0.81±0.13	0.74±0.16
**IL-16**	4.81±0.78^a,b^	2.61±0.36^c^	2.73±0.32^c^
**IL-17**	9.44±1.34^a^	9.14±1.46^a^	4.35±0.67^b,c^
**IL-18**	9.67±1.78^a,b^	1.245±0.16^c^	1.25±0.15^c^
**IL-27**	7.49±1.71^a,b^	6.13±1.31^a,c^	0.92±0.17^b,c^

Data are presented as mean ± SEM (standard error of the mean). Significantly higher mRNA expression in the TB group in contrast to the control group was detected for IL-10, IL-16, IL-17, IL-18, and IL-27. The mRNA expression of IL-10, IL-16, IL-18, and IL-27 was higher in the TB group than in the ID herniation group. a: Significant difference versus the control group (P<0.05). b: Significant difference versus the ID herniation group (P<0.05). c: Significant difference versus the TB group (P<0.05).

**Table 5:** The mRNA expression of the ILs in the different pathogenetic condition TB groups.

Gene	TB group			Control group (n = 10)
	Mild group (n = 8)	Moderate group (n = 11)	Servere group (n = 26)	
**IL-10**	8.41±1.21^a^	6.13±1.01^a^	2.16±0.23	2.15±0.43
**IL-12**	0.72±0.13	0.79±0.15	0.75±0.15	0.74±0.16
**IL-16**	3.11±0.19^a^	4.63±1.21^a^	5.23±1.01^a^	2.73±0.32
**IL-17**	4.78±0.79	9.21±2.11^a^	10.12±1.98^a^	4.35±0.67
**IL-18**	8.34±1.99^a^	9.11±1.91^a^	11.09±2.11^a^	1.25±0.15
**IL-27**	9.12±0.23^a^	8.45±1.09^a^	7.99±0.97^a^	0.92±0.17

Data are presented as mean ± SEM (standard error of the mean). Patients with more severe disease had significantly higher IL-16, IL-17, and IL-18 mRNA levels and significantly lower IL-10 and IL-27 mRNA levels. Mild group: VAS (0–4), CRP (<10 mg/L), ESR (<20 mm/h). Moderate group: VAS (5–7), CRP (10–30 mg/L), ESR (20–40 mm/h). Severe group: VAS (8–10), CRP (>30 mg/L), ESR (>40 mm/h). a: Significant differences versus the control group (P<0.05).

### Pairwise mRNA co-expression analysis

Multiple positive correlations were found in the TB and ID herniation groups concerning the mRNA expression of ILs. Specifically, in the TB group, IL-18 transcript levels were positively correlated with IL-16 and IL-17 expression, whereas IL-10 and IL-27 transcript levels were negatively correlated with IL-16, IL-17, and IL-18 levels. Furthermore, IL-16 and IL-17 mRNA were also found to be co-expressed in the TB group. Similarly, IL-10 and IL-27 mRNA were observed to be co-expressed in the TB group (Table 6).

**Table 6:** Correlation table demonstrating the pairwise co-expression profile of ILs in the ID herniation and TB group.

		IL-10	IL-12	IL-16	IL-17	IL-18	IL-27
**ID herniation group**							
	IL-10	−					
	IL-12	0	−				
	IL-16	(−)	0	−			
	IL-17	(−)	0	(+)	−		
	IL-18	(−)	0	(+)	(+)	−	
	IL-27	(+)	0	(−)	(−)	(−)	−
**TB group**							
	IL-10	−					
	IL-12	0	−				
	IL-16	(−)	0	−			
	IL-17	(−)	0	(++)	−		
	IL-18	(−)	0	(++)	(++)	−	
	IL-27	(++)	0	(−)	(−)	(−)	−

0 = not significant.

(+) = positive correlation, p<0.05.

(++) = positive correlation, p<0.001.

(−) = negative correlation, p<0.05.

(−) = negative correlation, p<0.001.

### Correlation analyses

Sex, employment, and the level of intervertebral disc herniation did not significantly affect the mRNA levels of the ILs included in the present study.

Age was positively correlated with elevated IL-18 mRNA levels in the TB and ID herniation groups (P = 0.023 and P = 0.007, respectively). Smoking habits were found to be positively correlated with the mRNA levels of IL-17 and negatively correlated with the mRNA levels of IL-10 in the TB group (P = 0.008 and P = 0.041, respectively).

**Figure 4:**
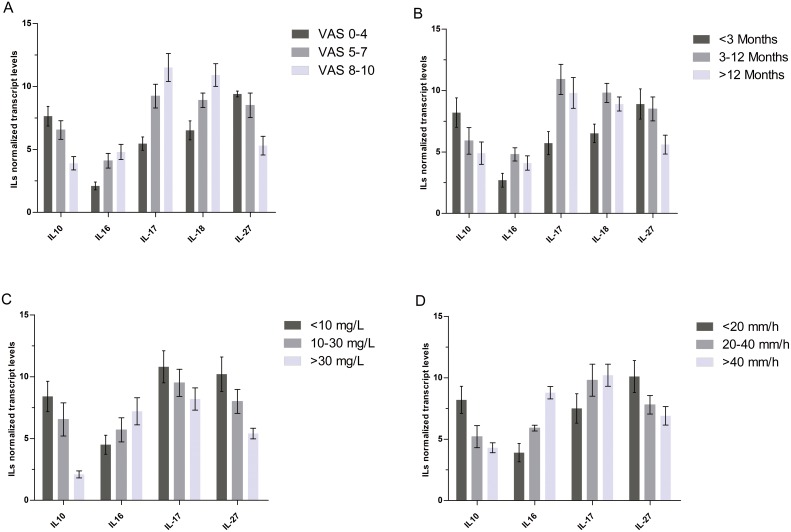
Normalized transcript levels of interleukins (ILs) in the TB group with respect to pain intensity (A), symptom duration (B), C-reactive protein (CRP) levels (C) and the erythrocyte sedimentation rate (ESR) (D). **A** illustrates that patients experiencing intense pain (visual analog scale [VAS] score = 8–10) exhibited higher IL-16, IL-17, and IL-18 mRNA levels than patients experiencing mild pain (VAS score = 0–4). Meanwhile, patients experiencing mild pain exhibited higher IL-10 and IL-27 mRNA levels than patients experiencing intense pain (P<0.05). **B** reveals that patients who experienced pain for 3–12 months exhibited significantly higher IL-16, IL-17, and IL-18 mRNA levels than those who experienced pain for <3 months. Conversely, patients who experienced pain for <3 months exhibited significantly higher IL-10 and IL-27 mRNA levels than those who experienced pain for 3–12 months (P<0.05). **C** indicates that the mRNA levels of IL-16 and IL-17 were significantly higher in patients with high CRP levels (>30 mg/L), whereas IL-10 and IL-27 levels were significantly higher in patients with low CRP levels (<10 mg/L) (P<0.005). **D** illustrates that the mRNA levels of IL-16 and IL-17 were significantly higher in patients with high ESRs (>40 mm/h). Conversely, IL-10 and IL-27 levels were significantly higher in patients with low ESRs (<20 mm/h) (P<0.005).

Pain intensity as assessed by a VAS significantly affected the transcript levels of IL-10, IL-16, IL-17, IL-18, and IL-27 (P = 0.009, P = 0.007, P = 0.034, P = 0.016, and P = 0.008, respectively). In particular, patients with intense pain (VAS score between 8 and 10) exhibited higher IL-16, IL-17, and IL-18 mRNA levels than patients with mild pain (VAS score between 0 and 4). Meanwhile, patients with mild pain exhibited higher IL-10 and IL-27 mRNA levels than patients with intense pain (Figure 4A).

The data revealed significant correlations between the duration of symptoms and the mRNA levels of IL-10, IL-16, IL-17, IL-18, and IL-27 in the TB group (P = 0.014, P = 0.033, P = 0.037, P = 0.006, and P = 0.009, respectively). Specifically, tissue samples obtained from patients who experienced pain for 3–12 months exhibited significantly higher IL-16, IL-17, and IL-18 mRNA levels than those who experienced pain for <3 months. Conversely, patients who experienced pain for <3 months exhibited significantly higher IL-10 and IL-27 mRNA levels than those who experienced pain for 3–12 months (Figure 4B).

CRP levels and the ESR were significantly correlated with the mRNA levels of IL-10, IL-16, IL-17, and IL-27 in the TB group (CRP: P = 0.018, P = 0.007, P = 0.024, and P = 0.009, respectively; ESR: P = 0.037, P = 0.041, P = 0.003, and P = 0.008, respectively). The mRNA levels of IL-16 and IL-17 were significantly higher in patients with high CRP levels (>30 mg/L), whereas the mRNA levels of IL-10 and IL-27 were significantly higher in patients with low CRP levels (<10 mg/L) (Figure 4C). Significantly higher IL-16 and IL-17 mRNA levels were observed in tissue samples obtained from patients with high ESRs (>40 mm/h) compared to those in patients with low ESRs (<20 mm/h). The mRNA levels of IL-10 and IL-27 were significantly higher in patients with low ESRs (<20 mm/h) (Figure 4D).

The data also illustrated significant associations among pain intensity, symptom duration, smoking, CRP levels, and ESRs. Specifically, CRP levels and ESRs were found to be positively correlated with the intensity of pain (P = 0.024 and P = 0.019, respectively). Furthermore, smoking was positively associated with pain intensity (P = 0.008).

## Discussion

TB is the leading cause of death from a curable infectious disease in China. The prevalence of active pulmonary tuberculosis in 2010 was 459 per 100,000 people [25]. In 3–5% of cases of active TB, osteolytic skeletal lesions develop; these occur mainly on the vertebrae [26], [27]. The typical bone lesion for TB is destruction of the anterior region of vertebral bodies and intervertebral discs with a subsequent collapse of the spine [26], [28].

Tuberculous vertebral bodies and intervertebral discs usually affect spinal stabilization, leading to deterioration of the patient’s condition [29]. In this study, investigations of spinal TB focused on the transcript levels of ILs in tuberculous intervertebral disc specimens, which were important for the inflammatory and immune mechanisms involved in the development of TB. Specifically, the mRNA levels of ILs were thoroughly analyzed by quantitative real-time PCR, and their correlation with the clinicopathological profile of patients with tuberculous intervertebral discs was examined.

There are many articles published on the expression levels of ILs in human TB [30]–[36]. Using enzyme-linked immunosorbent assay or PCR techniques, Valdes et al [30] examined the expression levels of IL-27 in patients with TB. The authors found that IL-27 levels were significantly higher in the tuberculous pleural effusions group. Similarly, Rovina et al [31] identified increased IL-18 activity in the pleural effusions of patients with TB compared to control specimens. Furthermore, Ibrahim et al [33] found that *M. tuberculosis* infection upregulates IL-16 expression and secretion in tuberculous pleural effusions. The underlying mechanisms of inflammatory and immune responses in patients with tuberculous spinal intervertebral discs remain poorly understood. ILs may play a central role in the pathology of spinal TB because of their ability to accelerate the production of matrix metalloproteinases that digest collagens I–IV [37].

In this study, significantly higher IL-10, IL-16, IL-17, IL-18, and IL-27 mRNA levels were observed in the TB group than in the control group. We speculate that these ILs may drive immune activation and extracellular matrix destruction in tuberculous spinal intervertebral discs. Furthermore, given the increased activity of IL-10, IL-16, IL-18, and IL-27 that has been observed in tuberculous intervertebral disc specimens compared to herniated disc specimens, the results suggest that the inflammatory and immune responses are more serious in a tuberculous intervertebral disc than in a herniated intervertebral disc.

The present study identified multiple correlations among IL-10, IL-16, IL-17, IL-18, and IL-27 mRNA levels in TB intervertebral disc specimens. Similar correlations were also observed in the ID herniation group. IL production in tuberculous disc tissue is influenced by cell-cell interactions, cell adhesion molecules, and cell-extracellular matrix interactions. For example, co-cultures of monocytes and endothelial cells produced more IL-8, monocyte chemoattractant protein 1, and macrophage inflammatory protein 1a than did either cell type alone [38]. Such cell-cell interactions can also downregulate certain ILs [38], probably depending on the type and stage of the involved inflammatory lesions. Multiple mechanisms are probably involved because IL expression is enhanced and suppressed by genetically controlled host factors, as well as factors from microorganisms themselves [39]. However, ILs also play a regulatory role in the interactions between macrophages and chondrocytes. In recent studies, IL-10 was reported to reduce joint swelling, cellular infiltration, pro-inflammatory cytokine production, and cartilage degradation in CIA in rats [40]. Therefore, the multiple correlations among IL-10, IL-16, IL-17, IL-18, and IL-27 levels are highly complex with interactive cascades of gene activation and suppression. The IL cascade hypothesis has played a crucial role in explaining the transcriptional co-expression of several ILs observed in tuberculous intervertebral disc tissue. The results also suggested a possible synergistic effect of multiple ILs in promoting inflammatory and immune reactions in patients with spinal TB. Better understanding of the immunopathogenic role of various ILs in the intervertebral disc destruction process will stimulate the development of more effective novel therapeutic strategies for spinal TB. Thus, further studies are necessary to elucidate the exact role of IL in the intervertebral disc destruction process of spinal TB.

Few studies have investigated the mRNA levels of IL-10, IL-16, IL-17, IL-18, and IL-27 in human tuberculous spinal intervertebral disc samples. This is the first study in which the transcript levels of ILs in human tuberculous spinal intervertebral disc samples were found to be directly associated with age, smoking habits, pain intensity, symptom duration, CRP levels, and ESRs. The results demonstrated that age was associated with increased transcript levels of IL-18. It is known that IL-18 promotes cartilage loss [41]. Aging has been found to promote the degradation of structural proteins, thus affecting IL-18 expression in tuberculous disc tissue. Cigarette smoking attenuates effector cytokine responses, alters disc matrix homeostasis, and impairs mycobacterial containment within infected human macrophages [42], [43]. Our research demonstrated that smoking habits were positively correlated with the mRNA levels of IL-17 and negatively correlated with the mRNA levels of IL-10 in the TB group. The aforementioned effects of cigarette smoking may alter inflammatory or anti-inflammatory reactions, lymphocyte infiltration, and cartilage degradation, thus affecting IL-17 and IL-10 expression in tuberculous disc tissue. Therefore, our results suggested that smoking plays multiple roles in the intervertebral disc destruction process of spinal TB.

Pain is considered an initiator factor of inflammatory events [44]. The duration of symptoms is believed to have a significant effect on the prognosis of spinal TB. The ESR and CRP levels are reliable parameters when evaluating the treatment and prognosis of spinal TB [45]. Our results indicated that pain intensity, symptom duration, CRP levels, and ESRs were associated with increased transcript levels of several ILs in the TB group. Moreover, IL-16 and IL-17 mRNA levels were found to be increased in patients with intense pain, high CRP levels, and high ESRs. Conversely, IL-10 and IL-27 mRNA levels were found to be decreased in these patients. IL-16 and IL-17 mRNA levels in patients were found to be correlated with the duration of pain. In theory, spinal TB initiates inflammatory and immune reactions via lymphocyte and mononuclear infiltration and IL upregulation, leading to inflammation. Active TB has been associated with an imbalance of the Th1/Th2 cytokine pattern. Effective induction of Th1 immunity is vital in the defense against *M. tuberculosis*. Furthermore, inflammatory cytokines, such as tumor necrosis factor-α and prostaglandin E2, are produced by infiltrating mononuclear cells immediately after the onset of spinal TB, further deterioration, and increased pain. In turn, the synergistic effects of multiple ILs induce the expression of other ILs. Therefore, a systemic inflammatory response is known to occur in patients with TB when these cytokines enter the systemic circulation, and as a result, the CRP blood level and ESR increase. Thus, we assume that four clinically relevant factors may affect inflammatory and immune responses by regulating the expression of ILs in tuberculous spinal intervertebral disc samples. Tuberculous spinal intervertebral disc pathogenesis is driven by the complex interplay between *M. tuberculosis* infection and the host’s inflammatory response. There are many inflammasome-related cytokines involved in inflammation in relation to the different stages of *M. tuberculosis* infection [46]. However, further studies are necessary to elucidate the exact role of ILs in the intervertebral disc destruction process of spinal TB.

## Conclusions

In conclusion, this study provides evidence of the molecular profile of ILs in tuberculous spinal intervertebral discs. IL-10, IL-16, IL-17, IL-18, and IL-27 are more strongly expressed in tuberculous spinal intervertebral disc tissue than in normal intervertebral disc tissue. This suggests that ILs play important roles in the intervertebral disc destruction associated with spinal TB. Multiple correlations among IL-10, IL-16, IL-17, IL-18, and IL-27 mRNA levels were found in tuberculous spinal disc tissue and imply a synergistic effect of the activity of these ILs in tuberculous spinal intervertebral discs. Smoking habits were found to have a positive correlation with the transcript levels of IL-17 and a negative correlation with the transcript levels of IL-10, suggesting multiple roles for smoking in the intervertebral discs destruction process of spinal TB.
